# Extracellular Sphingosine-1-Phosphate: A Novel Actor in Human Glioblastoma Stem Cell Survival

**DOI:** 10.1371/journal.pone.0068229

**Published:** 2013-06-24

**Authors:** Elena Riccitelli, Paola Giussani, Clara Di Vito, Giuseppe Condomitti, Cristina Tringali, Manuela Caroli, Rossella Galli, Paola Viani, Laura Riboni

**Affiliations:** 1 Department of Medical Biotechnology and Translational Medicine, University of Milan, LITA-Segrate, Milan, Italy; 2 IRCCS Cà Granda Foundation, Maggiore Policlinico Hospital, Milan, Italy; 3 Neural Stem Cell Biology Unit, Division of Regenerative Medicine Stem Cells and Gene Therapy, San Raffaele Scientiﬁc Institute, Milan, Italy; University of California, San Francisco, United States of America

## Abstract

Glioblastomas are the most frequent and aggressive intracranial neoplasms in humans, and despite advances and the introduction of the alkylating agent temozolomide in therapy have improved patient survival, resistance mechanisms limit benefits. Recent studies support that glioblastoma stem-like cells (GSCs), a cell subpopulation within the tumour, are involved in the aberrant expansion and therapy resistance properties of glioblastomas, through still unclear mechanisms. Emerging evidence suggests that sphingosine-1-phosphate (S1P) a potent onco-promoter able to act as extracellular signal, favours malignant and chemoresistance properties in GSCs. Notwithstanding, the origin of S1P in the GSC environment remains unknown. We investigated S1P metabolism, release, and role in cell survival properties of GSCs isolated from either U87-MG cell line or a primary culture of human glioblastoma. We show that both GSC models, grown as neurospheres and expressing GSC markers, are resistant to temozolomide, despite not expressing the DNA repair protein MGMT, a major contributor to temozolomide-resistance. Pulse experiments with labelled sphingosine revealed that both GSC types are able to rapidly phosphorylate the long-chain base, and that the newly produced S1P is efficiently degraded. Of relevance, we found that S1P was present in GSC extracellular medium, its level being significantly higher than in U87-MG cells, and that the extracellular/intracellular ratio of S1P was about ten-fold higher in GSCs. The activity of sphingosine kinases was undetectable in GSC media, suggesting that mechanisms of S1P transport to the extracellular environment are constitutive in GSCs. In addition we found that an inhibitor of S1P biosynthesis made GSCs sensitive to temozolomide (TMZ), and that exogenous S1P reverted this effect, thus involving extracellular S1P as a GSC survival signal in TMZ resistance. Altogether our data implicate for the first time GSCs as a pivotal source of extracellular S1P, which might act as an autocrine/paracrine signal contributing to their malignant properties.

## Introduction

Glioblastoma multiforme is the most frequent and aggressive primary central nervous system tumor in humans, with one of the worst survival rates of all the human cancers [1], due to a high proliferation rate, migrating and invasive properties, and resistance to current therapeutic intervention. Although the introduction of the alkylating agent temozolomide (TMZ) in glioblastoma therapy has improved patient survival, the prognosis of patients remains unfavorable [2]. Recent studies suggest that a subpopulation of cells, named glioblastoma stem cells (GSCs), exists within the tumor, and plays a crucial role in glioblastoma initiation, maintenance, and malignant behavior [3,4]. Of relevance, GSCs possess the ability to extensively self-renew and are capable of initiating the tumor upon orthotopic transplantation, giving rise to a heterogeneous population of cells such as those found in their parent tumors [5,6]. In addition, GSCs are thought to be responsible for maintaining these tumors after gross surgical resection and therapy, and are resistant to radiations and different cytotoxic drugs, including TMZ, the current mainstay of anti-glioma chemotherapy [7]. In spite different aberrations in GSCs may be involved in their intrinsic drug resistance [8–10], and the expression of the DNA repair protein O^6^-methylguanine-DNA methyltransferase (MGMT) appears a key factor strictly associated to their TMZ-resistance [11,12], our knowledge of the mechanisms underlying malignant and chemoresistance properties of GSCs remains limited. Thus, the molecular characterization of GSCs represents a critical step in defining glioblastoma properties, and may be essential in developing effective therapeutic strategies.

An increasing number of evidence indicates that the sphingoid molecule sphingosine-1-phosphate (S1P) is a potent bioactive lipid able to regulate a spectrum of essential cellular processes strictly related to cancer, such as proliferation, invasivity, survival and angiogenesis [13,14]. S1P is an intermediate of sphingolipid metabolism, and its cellular levels are finely regulated through the modulation of different enzymes responsible for its synthesis and degradation [15]. In cells, S1P is formed from sphingosine (Sph) and ATP in a reaction catalyzed by two isoenzymes, named sphingosine kinase 1 (SK1) and 2 (SK2) [16]. Once formed, S1P can be metabolized through two different pathways: the dephosphorylation back to Sph, and the irreversible cleavage to hexadecenal and phosphoethanolamine [17]. Accumulating evidence demonstrates that S1P plays an important role in the extracellular milieu, being secreted by some cell types, especially blood cells, but also endothelial and mast cells [18]. The finding that neurons and astrocytes can constitutively export S1P supports that also cells of the nervous system can be an origin of extracellular S1P [19,20]. Once released, S1P can act in an autocrine/paracrine manner, through interaction with specific transmembrane receptors (S1P_1-5_), coupled to different G-proteins and displaying tissue-specific expression patterns [21]. Through this interaction S1P can activate several signal transduction pathways, and thus elicit a variety of cell-specific responses controlling cell behaviour.

S1P has emerged as an onco-promoter molecule in different tumors, including glioblastomas [18,22,23]. In fact, it has been documented that S1P enhances proliferation, motility, invasiveness, and malignant behavior of glioblastoma cells [24,25]. In support to the importance of S1P in glioblastomas, SK1 is elevated in glioblastoma cell lines [26,27], is up regulated in glioma specimens in comparison with adjacent normal tissue, and its expression level correlates with the histological grade of the tumors and a poor patient survival [28,29]. In addition, other recent studies showed that SK1 inhibition causes apoptosis in TMZ-resistant glioma cells [30], and decreases growth and invasiveness of human GBM cells and of GBM cell xenograft growth in mice [31]. A further support to S1P as an important autocrine/paracrine messenger in glioblastomas emerges from the evidence that some glioma cell lines are able to release S1P in the extracellular milieu [27,32]. Moreover, it has been extensively demonstrated that glioblastoma cells commonly express different S1P receptors, all contributing to glioblastoma cell growth and invasion through distinct, but overlapping mechanisms [25,33,34].

Up to now, evidence on the role of S1P as an autocrine and/or paracrine factor modulating GSCs properties is scarce. Annabi and coworkers [35] reported that GSCs, isolated from a glioblastoma cell line, express S1P receptors and that exogenously added S1P promotes their migration and invasivity. Furthermore, Mora and colleagues [36] demonstrated that SK inhibition causes neurosphere dissociation, abolishes their growth, and promotes cell death in GSCs. Despite these studies suggest that S1P may act as an important mediator of GSCs properties, the ability of GSCs to produce and release S1P remains to be defined. Up to now, it is not known if GSCs are able to release S1P, or other cells within the tumor are the source of the extracellular S1P acting on GSCs. On these premises, the aim of this study was to investigate the ability of GSCs to produce and/or release S1P in the extracellular milieu and the role of this sphingoid molecule in GSC resistance to TMZ. By using GSCs derived from a human glioblastoma cell line, as well as those isolated from primary cultures of a human glioblastoma, this study demonstrate that GSCs can rapidly synthesize S1P, and efficiently release it extracellularly. In addition, we found that extracellular S1P acts as autocrine/paracrine signal involved in TMZ resistance, by acting as a survival molecule in GSCs.

## Materials and Methods

### Materials

All reagents were of analytical grade. Dulbecco’s modified Eagle’s medium (DMEM), penicillin, streptomycin, amphotericin B, epidermal growth factor (EGF), insulin, fatty acid free bovine serum albumin, aprotinin, leupeptin, pepstatin, bestatin, D-erythro-sphingosine, 3-[4,5-dimethylthiazol-2-yl]2,5-diphenyl tetrazolium bromide (MTT), Kodak Biomax film, and other common chemicals were from Sigma Aldrich (St. Louis, MO, USA). Fetal calf serum (FCS) was from EuroClone (Pero, Milan, Italy). Basic fibroblast growth factor (bFGF) was purchased from PeproTech (Rocky Hill, NJ, USA). B27 supplement and DMEM/F12 were obtained from Invitrogen (Carlsbad, CA, USA). RNeasy mini kit and RNase-free DNAse I were from Qiagen (Valencia, CA, USA), and iScript cDNA synthesis kit and SYBR green super mix from Biorad Laboratories (Hercules, CA, USA). TMZ was from Schering-Plough (Segrate, Milan, Italy). S1P was from Enzo Life Sciences (Farmingdale, NY, USA), and the SK inhibitor SKI from Echelon Biosciences Inc. (Salt Lake City, UT, USA). Goat anti-MGMT antibody and mouse anti-goat horseradish peroxidase-linked secondary antibody were from Santa Cruz Biotechnology (Santa Cruz, CA, USA). Rabbit anti-SK1 and anti-SK2 antibodies were purchased from Abcam (Cambridge, UK). Goat anti-rabbit horseradish peroxidase-linked secondary antibody, SuperSignal West Pico and West Femto Maximum Sensitivity Chemiluminescent Substrate were from Pierce Chemical Co. (Rockford, IL, USA). [^3^H]-D-erythro-sphingosine ([^3^H]-Sph) was purchased from PerkinElmer (Boston, MA, USA), and [γ-^32^P] ATP from GE Healthcare (Milan, Italy). High performance thin layer chromatography (HPTLC) silica gel plates and all solvents were from Merck (Darmstadt, Germany).

### Cell cultures

U87-MG human glioblastoma multiforme cell line was obtained from the Istituto Zooprofilattico Sperimentale della Lombardia e dell’ Emilia (Brescia, Italy) and cultured in DMEM containing 10% FCS (v/v), 2 mM L-glutamine, 100 units/ml penicillin, 100 µg/ml streptomycin and 0.25 µg/ml amphotericin B at 37°C in 5% CO_2_ humidified atmosphere.

GSCs derived from a human glioblastoma cell line (U–SC) were isolated in our laboratory from the stable U87-MG cell line through the use of selective medium composed of DMEM/F12, supplemented with 10 ng/ml bFGF, 20 ng/ml EGF, 5 µg/ml insulin, B27 supplement, 2 mM L-glutamine, 100 units/ml penicillin, 100 µg/ml streptomycin and 0.25 µg/ml amphotericin B [37–39]. To this purpose U87-MG cells were plated in 100 mm Petri dishes at 3 x 10^3^ cells/cm^2^ in 5 ml of selective medium and maintained at 37°C in a humidified atmosphere of 5% CO_2_ for one month. Every week the medium was replaced with fresh one, and the cells were counted to follow the selection process.

GSCs isolated from primary cultures of a human glioblastoma (L0627) were obtained in the R.G. laboratory from a post-surgery specimen of a primary human glioblastoma multiforme as previously reported [39]. L0627 were expanded *in vitro* in a selective medium consisting of DMEM/F12, and supplemented with 10 ng/ml bFGF and 20 ng/ml EGF.

### Real-Time PCR

Total RNA was isolated from U87-MG and U–SC cells with the RNeasy mini kit and treated with the RNase-free DNAse I. One microgram of RNA was reverse transcribed using the iScript cDNA synthesis kit according to manufacturer’s instructions. Real-Time PCR was performed using the iQ5 Real-Time PCR detection system (Biorad Laboratories, Hercules, CA, USA). Specific SYBR green expression assays (SYBR green super mix) for CD133, nestin and GAPDH were carried out. Simultaneous amplification of the target sequences was carried out as follows: 3 minutes at 95°C, 50 cycles 95°C 10 seconds, 58°C 40 seconds, 60°C 10 seconds and 1 cycle 60°C 3 minutes. Results were analyzed using the iQ5 optical system software (Biorad Laboratories, Hercules, CA, USA). Relative gene expression was determined using the 2^-ΔΔCt^ method [40]. Data were normalized to GAPDH expression (used as endogenous control) and U87-MG cells were used as calibrator.

### Cell treatments

Stock solutions were prepared by dissolving the following molecules as follows: TMZ and the SK inhibitor (SKI) in DMSO, and S1P in fatty acid free bovine serum albumin (4 mg/ml in PBS). Stock solutions were then diluted in fresh medium and administered to cells for the indicated periods of time. In parallel, cells were also incubated with vehicles as controls.

### Analysis of cell viability

Cell viability was determined by MTT assay. U87-MG and GSCs were seeded at 10^4^ and 2x10^4^ cells/cm^2^, respectively. The day after, cells were treated with different agents for the indicated periods of time. The medium was then replaced by MTT dissolved in fresh medium (0.8 mg/ml) for 4 hours. The formazan crystals were then solubilized in isopropanol/formic acid (95: 5 v/v) for 10 minutes and the absorbance (570 nm) was measured using a microplate reader (Wallack Multilabel Counter, PerkinElmer, Boston, MA, USA).

### Immunoblotting

*MGMT* - For MGMT evaluation, cells were lysed with 20 mM Tris-Cl pH 8.5, 1 mM EDTA, 5% glycerol, 1 mM β-mercaptoethanol, 0.1 mM PMSF in ethanol, in presence of protease inhibitors (10 µg/ml aprotinin, 5 µg/ml leupeptin, 5 µg/ml pepstatin, 3 µg/ml bestatin), as recently described [41]. Cell proteins were resolved by SDS-PAGE on 12% polyacrylamide gels and transferred onto nitrocellulose membranes. Membranes were then blocked in 5% milk using PBS-0,05%Tween 20, incubated for 1 hour with anti-MGMT primary antibody and finally with a mouse anti-goat horseradish peroxidase-linked secondary antibody. β-actin was used as loading control.

*SK* -For SK evaluation, cells were lysed with 20 mM HEPES pH 7.4, 50 mM NaCl,1 mM EGTA pH 8.1% Triton X100, 5 mM β-glycerophosphate, 2 mM sodium orthovanadate, 0.1 mM sodium pyrophosphate, 1 mM EDTA, in the presence of protease inhibitors (2 µg/ml each of aprotinin, leupeptin, and pepstatin). In order to evaluate SK1 expression, cell proteins were resolved by SDS-PAGE on 10% polyacrylamide gels and transferred onto nitrocellulose membranes. For SK2 expression, cell proteins were resolved by SDS-PAGE on 12.5% polyacrylamide gels and transferred onto PVDF membranes. Membranes were then blocked in 5% milk using TBS-Tween20 0.05%, incubated for 1 hour with anti-SK1, anti-SK2 primary antibodies and finally with goat anti-rabbit horseradish peroxidase-linked secondary antibody. GAPDH or β-actin were used as loading control. In all cases bound antibodies were visualized by ECL (SuperSignal West Pico or West Femto Maximum Sensitivity Chemiluminescent Substrate), and membranes were exposed to Kodak Biomax films.

### Metabolic studies

Cells were plated on 35 mm dishes in cell culture medium at 5x10^5^ cells/dish. At the time of the experiment, the medium was gently removed and cells were then pulsed with [^3^H]-Sph (20 nM, 0.4 µCi/ml) in S1P trapping medium [42]. Subsequently cells were harvested, total lipids extracted at 4°C with chloroform/methanol, and partitioned as previously reported [43]. After centrifugation, the upper alkaline aqueous phase, containing S1P, was evaporated under a nitrogen stream and counted for radioactivity by liquid scintillation.

Extracellular S1P was extracted from pulse medium and partially purified as previously described [19,20]. Briefly a two-step partitioning was performed, at first in alkaline conditions, and then a back extraction of the aqueous phase obtained was carried out in acidic conditions. The final organic phase, containing S1P, was evaporated under nitrogen stream. The aqueous phase, containing tritiated water produced from [^3^H]-S1P degradation, was purified by fractional distillation and counted for radioactivity [43]. The fractions containing cellular and extracellular S1P were submitted to HPTLC on silica gel plates, using n-butanol/acetic acid/water (3:1:1, v/v/v) as solvent system. Standard [^3^H]-S1P was chromatographed on the same plate, and used as internal standard. At the end of the chromatography, HPTLC plates were submitted to digital autoradiography (Beta-Imager 2000, Biospace, Paris, FR), and S1P was quantified by the beta vision analysis software (Biospace, Paris, FR).

### Sphingosine kinase activity in GSC-conditioned medium

GSCs (5 x 10^5^ cells/dish) were incubated at 37°C in fresh culture medium for different periods of time (30, 60 min, and 24 h). Media were then collected and concentrated (5000 x g, 1 h at 4°C). SK activity was then assayed using experimental conditions known to selectively favour SK1 or SK2 activity, as previously described [44–46], with some modifications. Briefly, the reaction mixture contained: concentrated medium (25-50 µl), 25 µM Sph as BSA-complex, 0.5 mM [γ-^32^P] ATP, 5 mM MgCl_2_ and SK buffer (20 mM Tris-Cl pH 7.4, 1 mM EDTA, 0.5 mM deoxypyridoxine, 15 mM NaF, 1 mM β-mercaptoethanol, 1 mM sodium orthovanadate, 40 mM sodium glycerophosphate, 10% glycerol and protease inhibitors) supplemented with either 0.5% Triton X100 (to assess SK1 activity), or 200 mM KCl (to assess SK2 activity). The mixture was incubated at 37°C for 30 minutes. S1P was then extracted [19] and resolved by HPTLC on silica gel plates, using 1-butanol/methanol/acetic acid/water (8:2:1:2, v/v/v/v) as solvent system. At the end of the chromatography, HPTLC plates were dried and labelled lipids visualized using autoradiography by overnight exposure to Kodak Biomax films. The radioactive spots corresponding to S1P were scraped from the plates, and counted for radioactivity by liquid scintillation. Background values were determined in negative controls where Sph was omitted in the reaction mixture.

### Statistical analysis

Results are expressed as means ± SD for at least three independent experiments. The statistical significance of the data was determined by the Student’s t-test. Differences were considered statistically significant at *P* < 0.05.

## Results

### Isolation and characterization of GSCs

**Figure 1:**
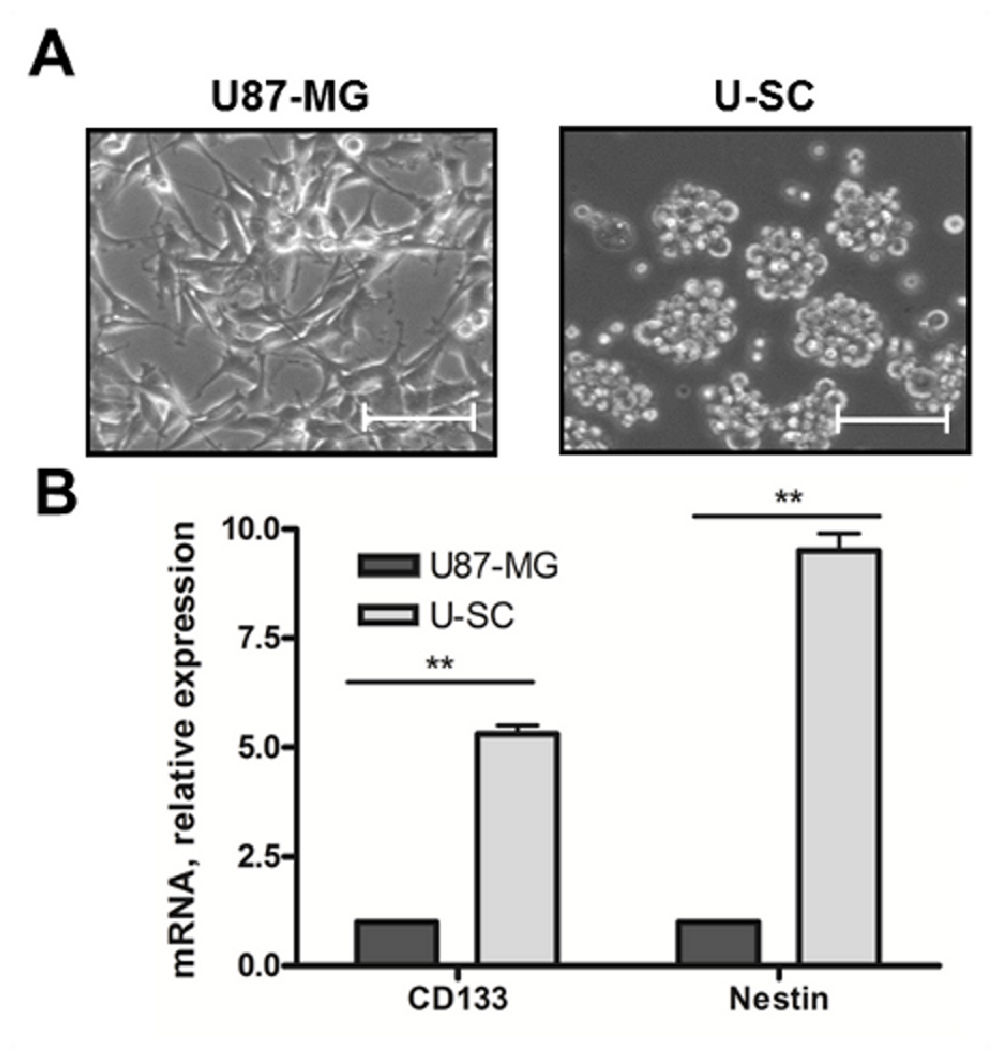
Characterization of cell models. (A) Representative images of U87-MG and U–SC morphology. Images were viewed on a contrast phase microscope and digital images were acquired (magnification, 10X; scale bar, 100 µm). (B) Expression of CD133 and nestin, assessed by Real-Time PCR. Results are expressed as fold-change relative to U87-MG. Values are the mean ± SD of three independent experiments. * p < 0.05; ** p <0.01 vs U87-MG cells.

GSCs were isolated from the human U87-MG glioblastoma multiforme cell line using a selective growth medium, which promotes the growth of the stem cell component (U–SC). Images acquired by phase-contrast microscopy showed obvious morphological differences between the two cell types (Figure 1A). U87-MG cells grew adherent to the surface of the growth support with a fibroblastoid-like morphology, characterized by the formation of numerous cytoplasmic extensions. Conversely, U–SC grew in suspension in the culture medium forming typical aggregates, called neurospheres, composed of 100-200 cells. It has been reported that brain tumor-derived cancer stem cells express high levels of the membrane glycoprotein CD133 and the cytoskeletal protein nestin [47,48]. In order to assess if the population of cells obtained by selective isolation was actually enriched in stem cells, we evaluated the expression of these stemness markers by real-time PCR. The results show that U–SC presented a 5.3 and 9.5 fold increase in CD133 and nestin expression, respectively when compared to U87-MG (Figure 1B). Thus, GSCs obtained from U87-MG are characterized by stem cells typical morphology and by increased expression of stemness markers, in agreement with previous reports [47,48]. As additional *in vitro* model for GSCs, we exploited L0627, previously isolated from a post-surgery specimen of a primary human glioblastoma multiforme. As U–SC, L0627 cells grew in suspension, efficiently formed neurospheres, and expressed high levels of the cancer stem cell markers CD133 and the adhesion molecule CD15, as previously reported [39,49]. Despite the cells’ true stem nature of glioma neurospheres remains debated, these GSCs reproduce the genotypic and phenotypic characteristics of glioblastomas more faithfully than standard glioma cell lines, and are considered an appropriate model of glioma [39,50].

### Survival properties of GSCs: evaluation of TMZ resistance

**Figure 2:**
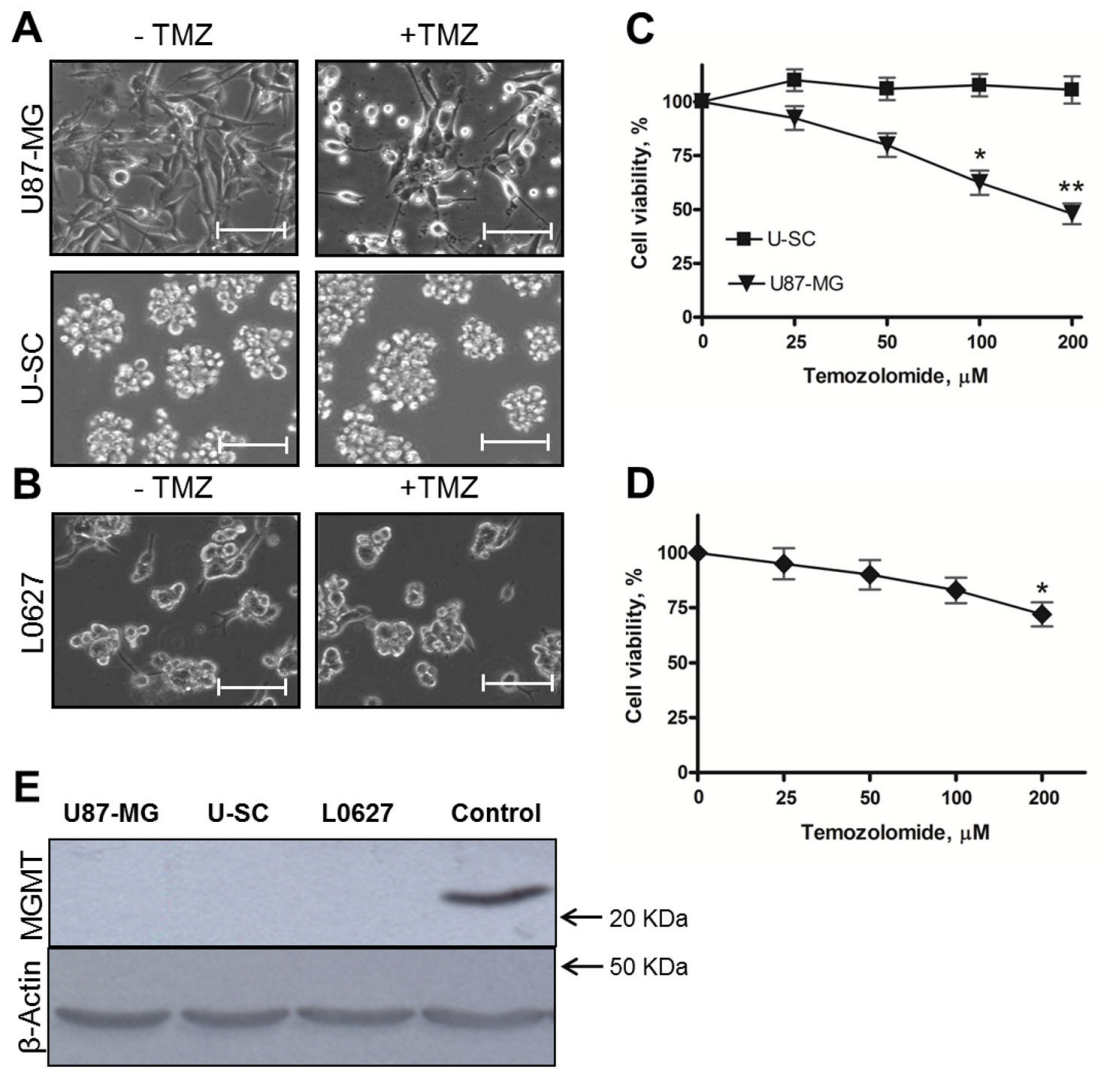
Effect of TMZ on cell survival. Representative images of (A) U87-MG and U–SC, and (B) L0627 morphology after 48 h treatment with vehicle (0.1% DMSO) or 100 µM TMZ (+ TMZ). Images were viewed on a phase contrast microscope, and digital images were acquired (magnification, 10X; scale bar, 100 µm). (C) U87-MG and U–SC, and (D) L0627 cells were exposed to different concentrations of TMZ or vehicle. Cell viability was assessed after 48 h of treatment by MTT assay. Results are expressed as percentage of cell viability with respect to vehicle-treated cells (100%). Data are the mean ± SD of three independent experiments. * p < 0.05; ** p < 0.01 vs vehicle-treated cells. (E) Cell lysates (60 µg of proteins) were analyzed by immunoblotting with anti-MGMT and anti-β-actin antibodies. 98G human glioblastoma cell lysates were used as control. The immunoblottings are representative of one out of three.

In order to analyze cell survival properties, increasing concentrations of TMZ (ranging from 25 to 200 µM) were used to treat U87-MG, U–SC and L0627 cells, and the cytotoxic effect was evaluated. Images obtained by phase contrast microscopy after 48 hours treatment with 100 µM TMZ showed that many of U87-MG cells were dead, in suspension, and those still adherent to the growth surface were strongly suffering, with a decreased cell size and the loss of normal fibroblastoid-like morphology (Figure 2A). In contrast, under the same conditions, U–SC and L0627 cells maintained unchanged their morphological features, with the typical formation of neurospheres (Figure 2A, B). These morphological pieces of evidence were supported by the analysis of cell viability by MTT assay. As shown in Figure 2C, the treatment with TMZ decreased U87-MG viability in a concentration-dependent manner. Indeed the treatment with 100 µM TMZ was associated with a cell survival of 60%, meanwhile that with 200 µM caused cell death in more than 50% of U87-MG cells. Conversely, the treatment with TMZ up to 200 µM did not cause any significant alterations in U–SC vitality (Figure 2C). Also L0627 viability did not significantly change after treatment with TMZ at 25-100 µM, the highest TMZ concentration (200 µM) being only partially effective (Figure 2D).

One of the best characterized mechanisms of TMZ resistance is the increased expression of the DNA repair protein MGMT [11]; therefore immunoblotting assays of MGMT were performed. As shown in Figure 2E, MGMT was not expressed not only in U87-MG, but also in U–SC and L0627.

### S1P biosynthesis and fates

**Figure 3:**
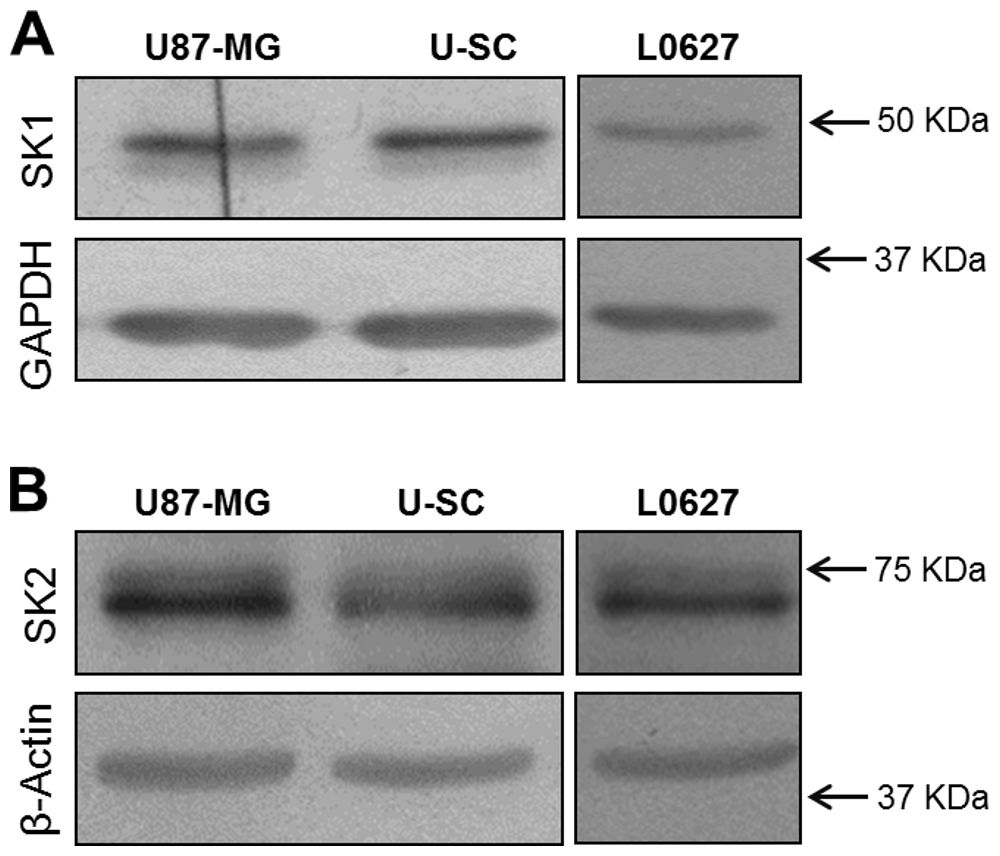
Expression of sphingosine kinases in U87-MG and glioma stem cells. Cell lysates (40 µg of proteins) were analyzed by immunoblotting with (A) anti-SK1 and anti-GAPDH antibodies, or (B) anti-SK2 and anti-β-actin antibodies. The immunoblottings are representative of one out of three.

An increasing number of evidence demonstrates that S1P plays an important role as an oncopromoter lipid, involved in the mechanisms of resistance to chemotherapy in different tumors, including glioblastoma [26,51]. On this base we first evaluated the expression of SK1 and SK2, the two known SK isoforms responsible for S1P synthesis. Immunoblotting assays demonstrated that U87-MG, U–SC, and L0627 cells all expressed both SK1 and SK2 (Figure 3).

**Figure 4:**
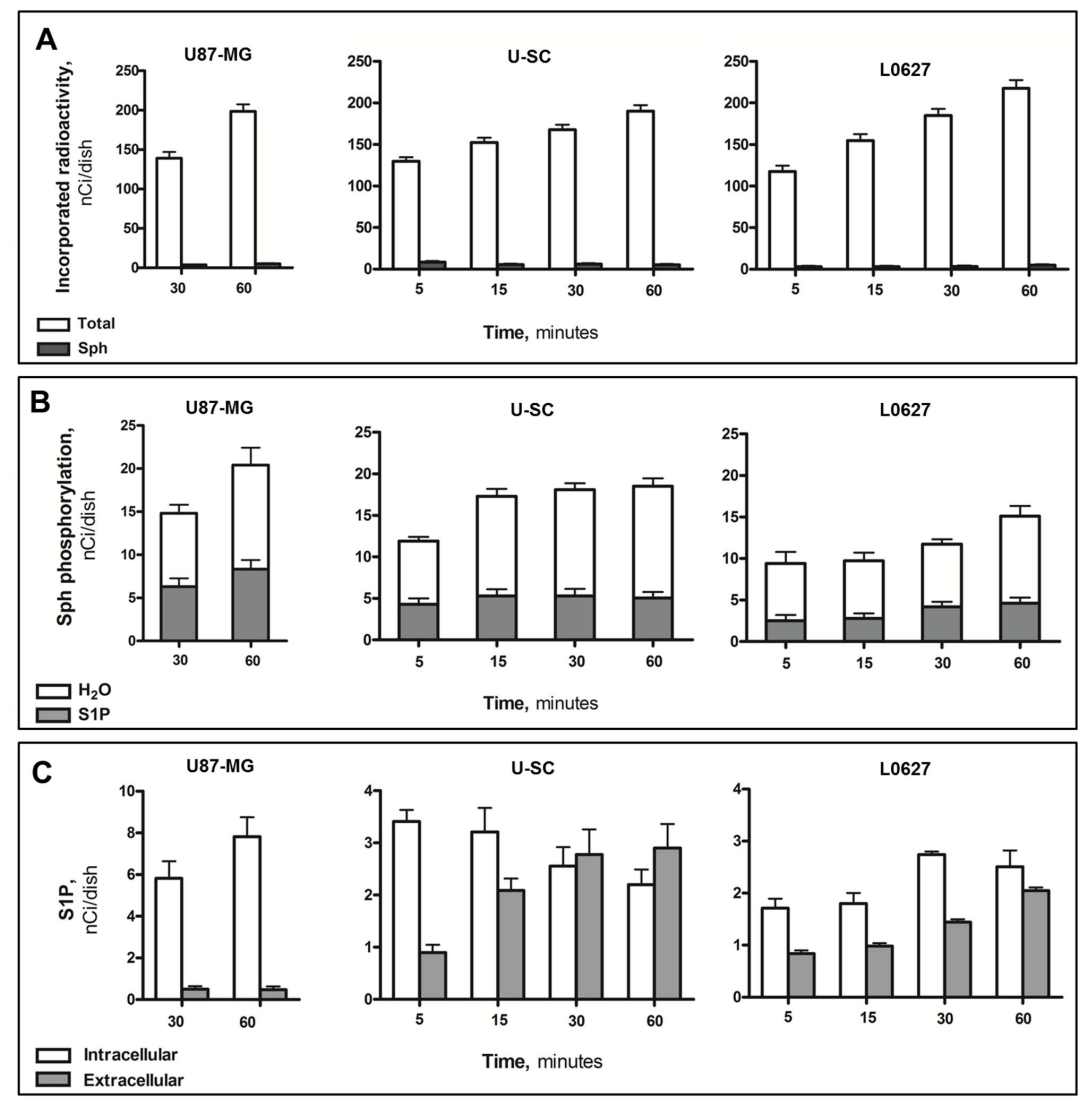
Biosynthesis and fates of S1P in U87-MG and glioma stem cells. U87-MG, U–SC and L0627 cells were pulsed with [^3^H]-sphingosine (Sph) for the indicated periods of time. At the end, cells and media were processed and analyzed as described in “Materials and methods”. Panel A, total incorporated radioactivity and intracellular Sph. Panel B, radioactivity associated to total Sph phosphorylation, total S1P and S1P degradation (^3^H _2_O). Panel C, radioactivity incorporated into intracellular and extracellular S1P. Data are the mean ± SD of at least three independent experiments.

In order to evaluate S1P production and metabolic fate, pulse experiments with tritiated Sph were performed. After short time pulse, the level of incorporated radioactivity was similar and increased in a time-dependent fashion in all cell models (Figure 4A). In these conditions, cellular [^3^H]-Sph represented less than 5% of the incorporated radioactivity in all cases, indicating that the cell types were able to rapidly incorporate and metabolize Sph (Figure 4A). In all cell models, the radioactivity associated to Sph phosphorylation (as the sum of S1P and tritiated water, its degradation product) increased with pulse time (Figure 4B). At all investigated times, tritiated water represented the bulk of Sph phosphorylation-associated radioactivity, and increased with pulse times (Figure 4B).

We then analyzed both cells and culture media for their [^3^H]-S1P content. As shown in Figure 4C, in U87-MG the levels of intracellular radioactive S1P increased in a time-dependent fashion, and about 3% of the radioactivity associated to Sph phosphorylation was detected extracellularly at both 30 and 60 min of pulse. In both GSC models the radioactivity associated with intracellular S1P was at least 2-fold lower than in U87-MG (Figure 4C). Of relevance, the amount of labeled S1P found in the extracellular milieu of both GSCs was markedly higher than that of U87-MG (Figure 4C).

**Figure 5:**
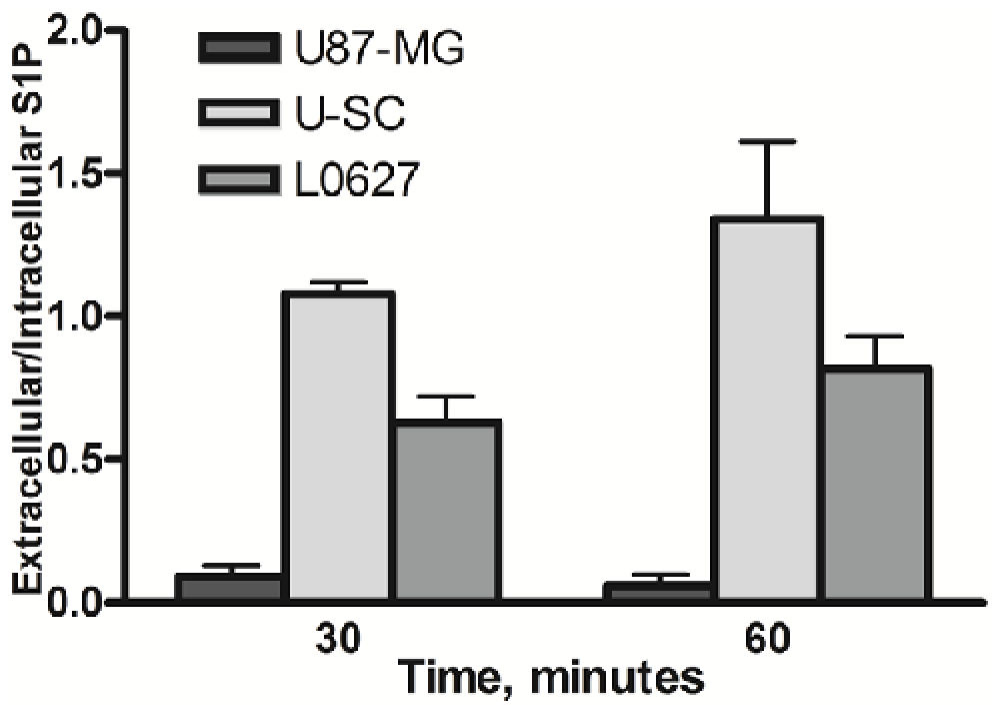
Extracellular/intracellular S1P ratio in U87-MG and glioma stem cells. U87-MG and both GSC types were pulsed with 20 nM [^3^H]-Sph (0.4 µCi/ml) for 30 or 60 minutes. At the end, S1P was extracted from cells and media and analyzed as described in “Materials and methods”. The extracellular/intracellular ratio of S1P-associated radioactivity is shown, as the mean ± SD of at least three independent experiments.

As shown in Figure 5, the ratio between extracellular and intracellular S1P was found to be about 10-fold higher in both GSCs than in U87-MG.

Prompted by these findings, we then hypothesized that the high amount of S1P retrieved in GSCs medium could be consequent to SK secretion in the extracellular milieu, as it occurs in some cell types [44,52,53]. We then evaluated SK1 and 2 activities in cell-conditioned medium. In the used experimental conditions, the activity of both SK isoforms was undetectable in the medium from all cell types (data not shown).

### Role of S1P in GSCs survival properties

**Figure 6:**
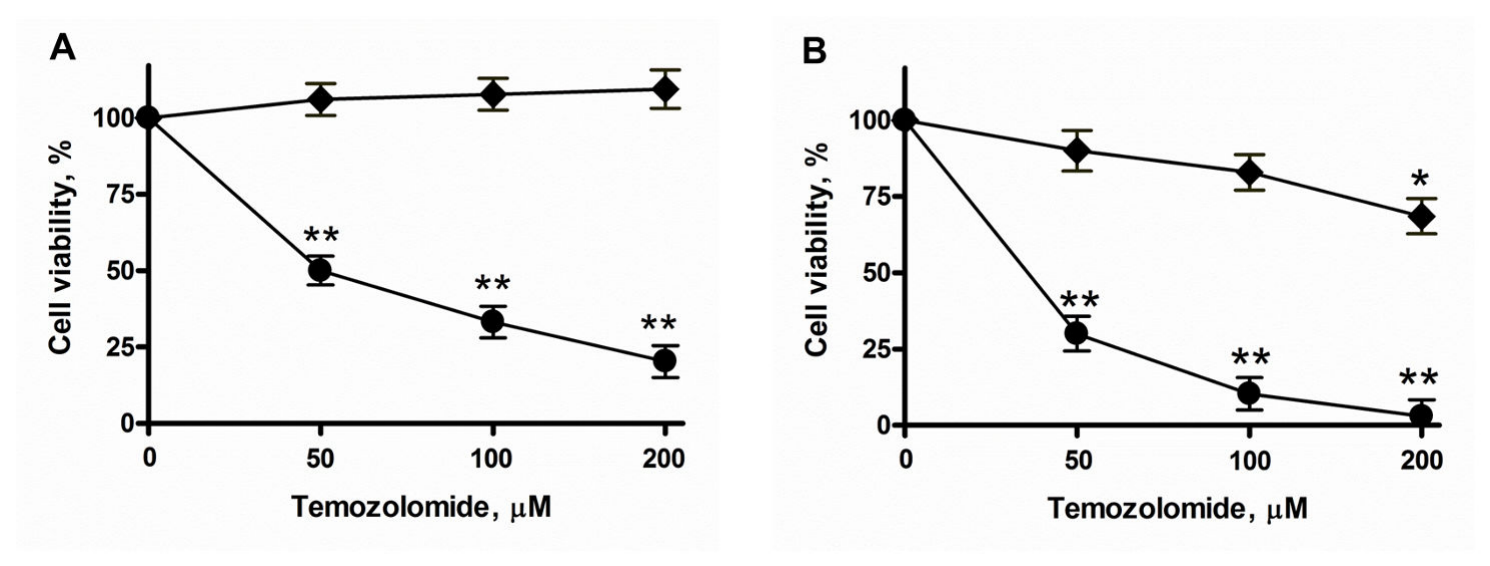
Role of S1P in GSC survival properties. U–SC (A) and L0627 cells (B) were exposed to 50-200 µM of TMZ alone (♦) or in combination with 4 µM SKI (●). After 48 hours of treatment, cell viability was assessed by the MTT assay. Results are expressed as percentage of cell viability with respect to vehicle-treated cells (♦) or cells treated with SKI alone (●) (100%). Data are the mean ± SD of three independent experiments. * p < 0.05; ** p < 0.01 vs vehicle-treated cells.

Stimulated by these results, we then investigated the possible effect of S1P in GSC sensitivity to TMZ. First, we evaluated the effect of SK inhibition by administrating the SKI inhibitor together with TMZ. We found that the co-treatment with TMZ and SKI (both at sub-toxic concentrations) significantly reduced cell viability (Figure 6). In particular, in GSCs treated with TMZ in the presence of SKI, cell viability was decreased by 70-90%, compared to cells treated with TMZ alone. Taken together these data indicate that the inhibition of SKs increases GSC sensitivity to TMZ toxicity, thus reducing GSC refractoriness to TMZ.

**Figure 7:**
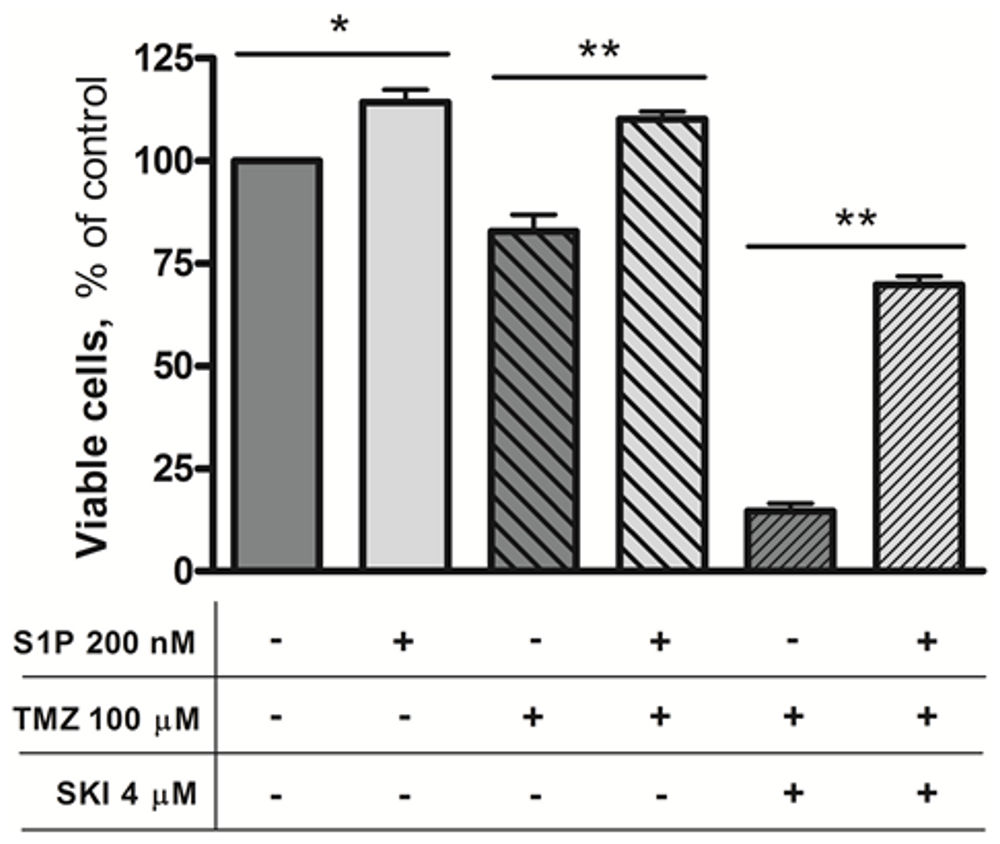
Effect of extracellular S1P on GSC resistance to TMZ. L0627 cells were exposed to 200 nM S1P, 100 µM TMZ, and 4 µM SKI, separately or in combination, as indicated. When used, after 24 hours 200 nM S1P was added again. After 48 hours of treatment, cell viability was measured by the MTT assay. Results are expressed as percentage of viable cells with respect to vehicle-treated ones (100%). Data are the mean ± SD of at least three independent experiments. * p < 0.05; ** p < 0.01 vs vehicle-treated cells.

Subsequently, in order to assess the effect of extracellular S1P in GSC TMZ resistance, we evaluated the effect of nanomolar concentrations of S1P on cell survival in the presence of TMZ and SKI, separately or in combination. As shown in Figure 7, the results of these experiments revealed that in GSCs the administration of exogenous S1P to control and TMZ-treated cells significantly increased the number of viable cells, supporting a growth- and survival-promoting effect of the phosphorylated Sph. Finally and remarkably, the treatment with S1P resulted in a 5-fold increase in viable cells, and thus in the reversion of the toxic effect of SKI-TMZ co-treatment.

## Discussion

In human glioblastomas, the presence of GSCs has been related to a poor prognosis, and these cells appear the responsible for glioma recurrences due to their chemo- and radio-resistance [54,55]. Thus it is conceivable that therapies targeting this cell population will provide a new approach in the treatment of glioblastomas. It is in this context that this research was performed, with the aim to contribute to the understanding of the pathways crucial for GSC survival properties, and focusing on the role of S1P as possible survival mediator in GSCs. We used GSCs isolated either from the human U87-MG glioblastoma cell line, or from a post-surgery specimen of a primary human glioblastomas. Despite the cultured GSC as neurosphere represents an in vitro model of GSCs, they are recognized as an appropriate model of gliomas [37–39]. We first obtained evidence that concentrations of TMZ that were toxic in human U87-MG cells, had no or only limited effects on GSCs viability. Of relevance, our data demonstrate that GSCs were resistant to 100-200 µM TMZ, that is to concentrations higher than those observed in patients submitted to TMZ chemotherapy. Indeed, in the plasma and cerebrospinal fluid of glioblastoma patients, the level of this drug was reported to range from 0.5 to 70 µM, and from 0.8 to 10 µM, respectively [56]. Thus, the used GSC models were resistant to therapeutic doses of TMZ, in agreement with the hypothesis that GSCs play a pivotal role in glioblastoma chemotherapy resistance [5]. Notably, the expression of the DNA repair protein MGMT, a major contributor to TMZ resistance [57], was undetectable in both GSC populations, indicating that mechanisms other than MGMT are involved in TMZ resistance of these cells.

Further experiments demonstrated that GSCs expressed both SK isoforms, and pulse studies showed that this cells were able to efficiently synthesize and degrade S1P, indicating a very rapid Sph metabolism. The analyses of the pulse media led us to obtain the major finding of this study, i.e. that GSCs can rapidly and efficiently produce S1P extracellularly, and thus can contribute to S1P levels in the extracellular environment. In particular, extracellular S1P in GSCs was found significantly higher than in U87-MG, and this difference resulted in an extracellular/intracellular S1P ratio of about 1:1 in both GSCs, that is at least 10-fold higher than that of U87-MG. These findings led us to hypothesize that the relevant amount of S1P in GSC medium could be consequent to SK secretion in the extracellular milieu, as it occurs in other cell types [44,52,53]. However, investigations on the SK presence in GSC medium led us to exclude this hypothesis, and suggest the involvement of a protein-mediated transport in the S1P export from GSCs. Since different ABC transporters have been implicated in S1P release [21], it will be of relevance to identify their potential involvement in the efficient release of S1P into the extracellular milieu in GSCs. Studies are in progress to identify this important mechanism.

It has been shown that the up-regulation of the SK isoform SK1 correlate with shorter survival time of patients with GBM [28,29]. In addition, other recent studies showed that SK1 inhibition causes apoptosis in TMZ-resistant glioma cells [30]. In spite both SK1 and SK2 were found to be expressed in GSCs, and these kinases appear to have at least some overlapping roles, it is tempting to speculate that SK1 might be involved in the intracellular generation and subsequent release of S1P in GSC.

The finding that GSCs can export S1P in the extracellular environment is of relevance for different reasons. First, it is acknowledged that S1P can act as a potent onco-promoter molecule in glioblastomas [24,25,33], and GSCs appear to contribute to the extracellular level of this messenger, thus favoring tumor progression. Second, emerging pieces of evidence demonstrate that GSCs do express S1P receptors, and that S1P administration promotes their proliferation, migration, and invasivity [35,36], supporting a role of extracellular S1P in GSC properties. Last, but not least, the evidence of the efficient production, and extracellular release of S1P by GSCs implies its role as an autocrine/paracrine signal of the GSC microenvironment. In this context, it is worth noting that neural stem cells (NSCs) appear to depend to other cells for the presence of S1P in their extracellular milieu. Indeed, it was shown that extracellular S1P is a potent chemo-attractant messenger in NSCs, but these cells are not able to release S1P in their external environment [58]. Thus it emerges that, differently from NSCs, GSCs exhibit the ability to enrich their extracellular milieu of S1P, representing an important source of this onco-promoter mediator in the extracellular environment.

The ability of GSCs to release S1P extracellularly is most likely related to their expression of S1P receptors, and to the pro-tumor effects of S1P on these cells. In agreement with this hypothesis, and with the role of S1P as an important oncopromoter lipid involved in chemotherapy resistance [18,22,23], we found that S1P plays a key role in GSCs resistance to TMZ. Indeed we first observed that the inhibition of S1P biosynthesis, through the SKI inhibitor, made GSCs sensitive to TMZ. Moreover, exogenous S1P, alone or in combination with TMZ, was able to revert the cytotoxic effect of the co-treatment with TMZ and the SKI inhibitor, promoting GSC survival. It is worth noting that the SKI was effective in promoting TMZ toxicity even at drug concentrations found *in vivo.*

Data from the literature provided evidence that CD133^+^ cells are much more responsive to extracellular S1P than are their parental counterparts, and differential S1P receptor expression contribute to this feature [35]. Since S1P1 promotes cell survival, and its expression is increased in CD133^+^ U87 cells, as well as in experimental and intracranial glioblastomas [35], it appears reasonable that S1P1 might be involved in the pro-survival effect of extracellular S1P in TMZ-treated GSCs. In support, a recent study [59] published while this work was in progress reported on the effect on GSC viability of FTY720, a S1P agonist which bears structural similarity to S1P and binds to S1P receptors, and its binding induces internalization and degradation of the S1P1 receptor, resulting in prolonged down-regulation of this receptor [60]. It was found that *in vitro* administration of FTY720 to GSCs decreased cell viability and acted synergistically with TMZ in promoting cytotoxicity [59] Moreover, the same study showed that FTY720 decreased GSCs invasiveness in nude mouse brains and promoted mouse survival.

In conclusion, this study demonstrates the pivotal role of extracellular S1P as a mediator of TMZ resistance in GSCs. Altogether our data implicate, for the first time, GSCs as an important S1P source in the extracellular microenvironment, which acts as an autocrine/paracrine signal contributing to their survival properties. Finally, the data presented herein may serve useful not only in the study of signal pathways involved in TMZ-induced apoptosis, but also in the understanding the possible significance of S1P and its modulators in the treatment of malignant glial tumors.
